# Probing genomic diversity and evolution of *Streptococcus suis *serotype 2 by NimbleGen tiling arrays

**DOI:** 10.1186/1471-2164-12-219

**Published:** 2011-05-10

**Authors:** Zuowei Wu, Ming Li, Changjun Wang, Jing Li, Na Lu, Ruifen Zhang, Yongqiang Jiang, Ruifu Yang, Cuihua Liu, Hui Liao, George F Gao, Jiaqi Tang, Baoli Zhu

**Affiliations:** 1 CAS Key Laboratory of Pathogenic Microbiology and Immunology, Institute of Microbiology, Chinese Academy of Sciences, Beijing, China; 2 Department of Microbiology, Third Military Medical University, Chongqing, China; 3 Department of Epidemiology, Research Institute for Medicine of Nanjing Command, Nanjing, China; 4 Institute of Microbiology and Epidemiology, Academy of Military Medical Sciences, Beijing, China; 5 Beijing Institutes of Life Science, Chinese Academy of Sciences, Beijing, China

## Abstract

**Background:**

Our previous studies revealed that a new disease form of streptococcal toxic shock syndrome (STSS) is associated with specific *Streptococcus suis *serotype 2 (SS2) strains. To achieve a better understanding of the pathogenicity and evolution of SS2 at the whole-genome level, comparative genomic analysis of 18 SS2 strains, selected on the basis of virulence and geographic origin, was performed using NimbleGen tiling arrays.

**Results:**

Our results demonstrate that SS2 isolates have highly divergent genomes. The 89K pathogenicity island (PAI), which has been previously recognized as unique to the Chinese epidemic strains causing STSS, was partially included in some other virulent and avirulent strains. The ABC-type transport systems, encoded by 89K, were hypothesized to greatly contribute to the catastrophic features of STSS. Moreover, we identified many polymorphisms in genes encoding candidate or known virulence factors, such as PlcR, lipase, sortases, the pilus-associated proteins, and the response regulator RevS and CtsR. On the basis of analysis of regions of differences (RDs) across the entire genome for the 18 selected SS2 strains, a model of microevolution for these strains is proposed, which provides clues into *Streptococcus *pathogenicity and evolution.

**Conclusions:**

Our deep comparative genomic analysis of the 89K PAI present in the genome of SS2 strains revealed details into how some virulent strains acquired genes that may contribute to STSS, which may lead to better environmental monitoring of epidemic SS2 strains.

## Background

*Streptococcus suis *serotype 2 (*S. suis *2, SS2) is an important zoonotic pathogen that causes severe porcine infectious diseases, including arthritis, meningitis, and pneumonia [1-3]. Virulent strains of SS2 can also be transmitted to humans (especially abattoir workers and pork handlers) by direct contact, causing meningitis, permanent hearing loss, septic shock, and even death. Two large-scale outbreaks of severe SS2 epidemics occurred in China in 1998 and 2005, causing great economic losses in the swine industry. These two outbreaks also posed serious public health risks from the newly emerging streptococcal toxin shock syndrome (STSS), which claimed 52 lives [4]. Over the past decade, considerable attention has been given to the study of virulence factors (e.g., CPS, MRP, EF, and suilysin) and the pathogen-host interaction in this emerging pathogen. However, comparative studies at the whole-genome level had little done to decipher the evolutionary aspects by which the virulence and environmental adaptation of SS2 are shaped.

To shed light on the evolution of pathogenicity and potential genomic polymorphisms of SS2, several virulent strains were subjected to whole-genome sequencing and comparative genomic studies. Comparative analysis of the whole-genomic DNA sequence of the European *S. suis *strain P1/7 (by the Sanger Institute) and two representative highly virulent strains (98HAH12 and 05ZYH33) isolated from STSS patients during the two epidemic outbreaks in China uncovered a candidate pathogenicity island (PAI) named 89K, which has been confirmed to undergo horizontal gene transfer (HGT) by our recent work [5]. Further analysis based on PCR amplification revealed that 89K exclusively present in the epidemic strains in these two Chinese SS2 outbreaks but not in other domestic clinical isolates or international virulent strains [6]. However, analysis of the unfinished genomic sequence of SS2 strain 89/1591 (by the DOE Joint Genome Institute) revealed that a partial 89K sequence (~30 kb) is present in this typical North American virulent strain. Similarly, results from a recently published work suggest that *S. suis *strain BM407, which was isolated from a human meningitis case in Vietnam in 2004, contains two regions with extended similarity to 89K [7]. These findings led us to hypothesize that the genome of SS2 would be highly polymorphic among different strains.

In this study, we employed the comparative genome re-sequencing (CGS) approach developed by Roche NimbleGen Systems to investigate genomic diversity in a collection of 18 SS2 strains, including isolates from the two outbreaks in China, other virulent strains from China (isolated before these outbreaks), virulent strains from European countries, and several avirulent strains. Although CGS cannot identify recently gained genes due to technical limitations, the DNA microarray-based comparative genome sequencing technique allows high resolution detection of sequence polymorphisms based on a reference genome [8,9]. Using this technology, we identified a number of novel genetic polymorphisms in SS2 strains and several candidate virulence factors that may contribute to STSS. Our results provide new insight into the virulence mechanisms and genome dynamics of SS2, which will help to elucidate the evolution of SS2 strains and better monitor the incidence and spread of epidemic strains.

## Results

We used NimbleGen tiling arrays to analyze the genomic variability of 18 SS2 isolates (Table 1), including five strains (ZYH214, ZYH215, ZYH354-1, Habb, and 98T003) isolated from STSS patients during the two Chinese SS2 outbreaks, two strains (JR and JZD-1) isolated from dead pigs during the 2005 outbreak in China, eight virulent strains isolated from China before the 1998 outbreak (strains 606, 1940, and 1941) or from other countries (strains 607, SS2-N, S735, 8011, and S10), and three avirulent strains (05HAS68, ZF, and T15). The complete genome sequence of 05ZYH33 (GenBank accession number NC_009442), a highly virulent strain isolated from a Chinese STSS patient during the Sichuan outbreak in 2005 [4], is available and was used as the common reference strain for each CGS analysis.

**Table 1:** Characteristics of the *S. suis *strains used in this study

Strain	Origin (Year)	Location	Virulence
05ZYH33	STSS patient, 2005	Sichuan, China	Virulent
ZYH214	STSS patient, 2005	Sichuan, China	Virulent
ZYH215	STSS patient, 2005	Sichuan, China	Virulent
JR	Diseased swine, 2005	Jiangsu, China	Virulent
JZD-1	Diseased swine, 2005	Jiangxi, China	Virulent
ZYH354-1	STSS patient, 2005	Sichuan, China	Virulent
Habb	STSS patient, 1998	Jiangsu, China	Virulent
98T003	STSS patient, 1998	Jiangsu, China	Virulent
606	Diseased swine, 1980s	China	Virulent
607	Diseased swine, 1980s	Japan	Virulent
1940	Diseased swine, 1980s	China	Virulent
1941	Diseased swine, 1980s	China	Virulent
SS2-N	Diseased swine, 1996	Germany	Virulent
S735	Diseased swine, 1963	The Netherlands	Virulent
8011	Swine, 1996	The Netherlands	Virulent
S10	Swine, ?	The Netherlands	Virulent
05HAS68	Healthy swine, 2005	Jiangsu, China	Avirulent
ZF	Healthy swine, 2006	Jiangsu, China	Avirulent
T15	Healthy swine, ?	The Netherlands	Avirulent

### Phylogenetic profiling of the SS2 strains

**Figure 1:**
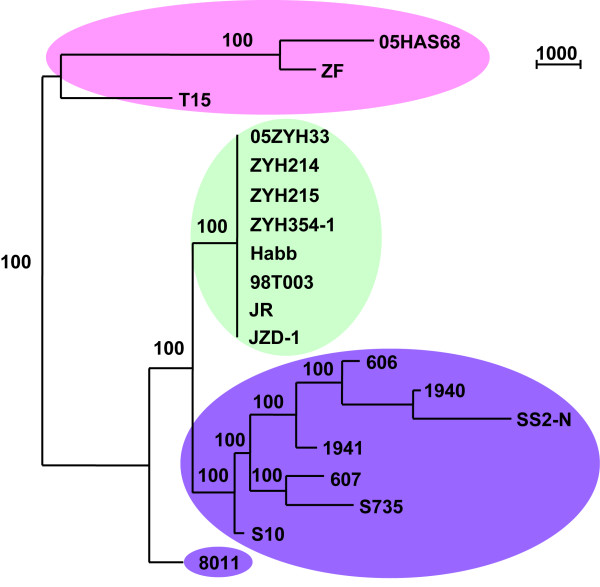
**Phylogenetic profiling (maximum parsimony tree) of SS2 strains based on mutation mapping results**. The three avirulent strains are labeled in pink, the virulent strains isolated in the two STSS outbreaks in China are labeled in light green, and the virulent strains collected before the two STSS outbreaks are labeled in purple.

A total of 299,469 29-mer probes were synthesized according to the genome sequence of 05ZYH33 to map the genomic variations of different SS2 strains. The NimbleGen algorithms assign each probe a '1'or '0' status based on the probe signal intensity ratio (reference/test) to reflect the sequence similarity of the corresponding region. A phylogenetic tree was then drawn based on the probe status of the entire probe set from the various SS2 strains (Figure 1). Consequently, the SS2 strains analyzed could be clustered into three major groups, the eight virulent strains isolated from the two STSS outbreaks in China were grouped together, and their genomes were highly similar to one another. The virulent strains isolated from European, Japan, and China before the two Chinese STSS outbreaks were grouped together, except the Netherlands 8011 strain. Considerable genome variation, however, exists among members of this group. Strain 8011 formed a single clade separated from the other virulent and avirulent strains. The three avirulent strains were clustered into the third group, and the two isolates from China (05HAS68 and ZF) were more closely related to each other than to the avirulent strain T15 isolated from the Netherlands. The results of phylogenetic profiling revealed that the virulent strains isolated from the two STSS outbreaks in China are highly homologous, and genomic polymorphisms were significant in SS2 populations. In general, the classification of these 18 SS2 strains based on genome similarity and variation, as revealed by NimbleGen analysis, is in good agreement with the epidemiological history and differential virulence of these strains.

### Polymorphisms in the 89K PAI

**Figure 2:**
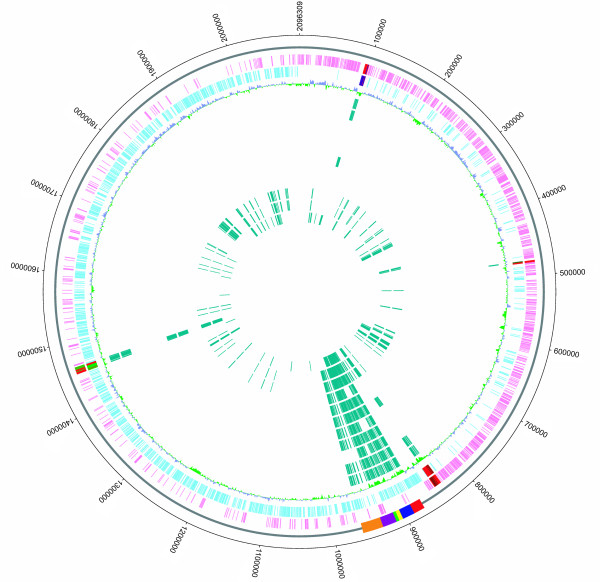
**Circular representation of the reference genome 05ZYH33 and comparative genomic hybridizations using NimbleGen tilling arrays**. The outer circle shows the genome scale. Rectangles on the second circle denote locations of the 89 K PAI, including Region I (red), Region II (blue), Region III (yellow), Region IV (green), Region V (purple), and Region VI (orange). The third and fourth circles display predicted coding regions on the plus and minus strands, respectively. The fifth circle shows the GC content (in a 1-kb window and 100-bp incremental shift). Values > 41.1% (average) are plotted outward (blue) and values < 41.1% are inward (green). Inner circles show all of the deleted genes identified in the 11 SS2 strains, with SS2-N, S735, 8011, S10, 606, 607, 1940, 1941, 68, ZF, and T15 ordered from the sixth to the sixteenth circle, respectively.

**Figure 3:**
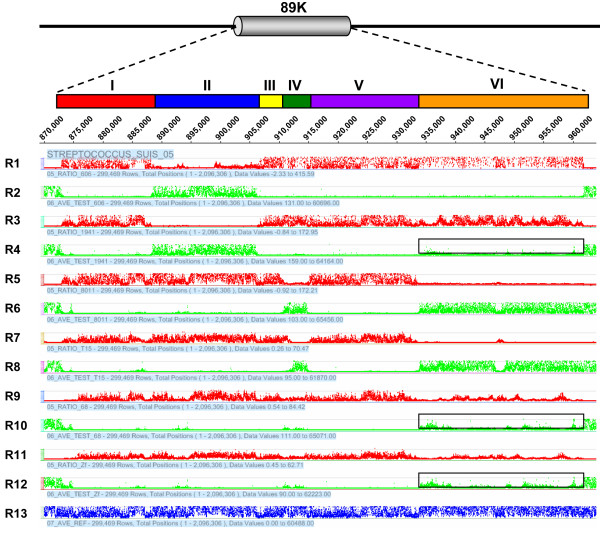
**Polymorphisms in the 89 K PAI**. The chromosomal location of the 89 K PAI in strain 05ZYH33 is shown and divided into six regions. The R2, R4, R6, R8, R10, and R12 plots (green) show the hybridization signal intensity for strains 606, 1941, 8011, T15, 68, and ZF, respectively. Ratios of hybridization signal intensity between each tested strain and the 05ZYH33 reference strain are calculated at each base position and are shown in the R1, R3, R5, R7, R9, and R11 ratio plots in the same order (red). The R13 plot (blue) shows the hybridization signal intensity for the reference strain. Regions of low signal intensity of strains 05HAS68, ZF, and 1941 are highlighted with black rectangles.

**Figure 4:**
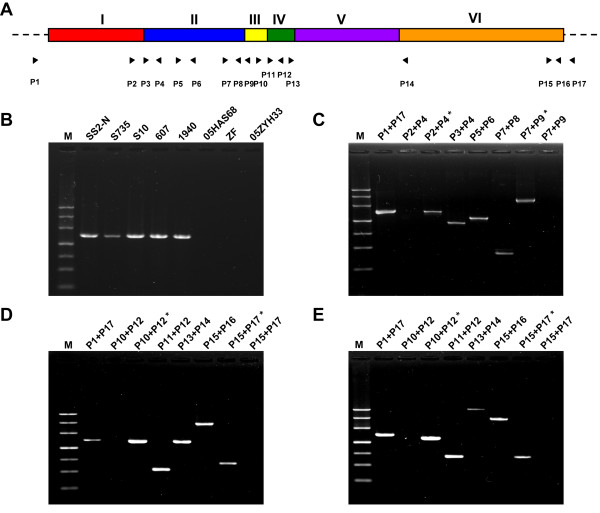
**PCR confirmation of the 89 K polymorphisms in the SS2 strains**. **(A) **Schematic representation of SS2 genomes with 89 K and relevant primers (shown as black triangles). **(B) **PCR detection of 89 K with primers P1&P17. **(C) **PCR detection of 89 K in strains 606 and 1941. **(D) **PCR detection of 89 K in 8011. **(E) **PCR detection of 89 K in T15. The primer combinations used in the PCR analysis are marked on the lanes. The asterisk represents strain 05ZYH33 being used as the template. M, the DNA marker, from (top to bottom) 4.5, 3, 2, 1.2, 0.8, 0.5, and 0.2 kb, respectively.

In previous studies, the so-called 89K genomic island of strain 05ZYH33 was considered a candidate PAI of virulent SS2 strains responsible for the two STSS outbreaks in China and was unique to STSS-causing virulent isolates collected since 1998 [6]. The 89K island is ~89-kb in length and composed of 80 predicted genes (SSU05_0903~0982) in the genome of 05ZYH33 (additional file 1). Consistently, our CGS and PCR analysis revealed that all eight virulent strains isolated from the two Chinese outbreaks contain the entire 89K fragment, whereas the international virulent strains SS2-N, S735, S10, and 607, as well as one strain (1940) isolated in China before the 1998 outbreak, completely lacks this element (Figure 2). However, the 89K PAI displays a considerable number of polymorphisms in the other SS2 strains (Figure 2, 3, &4). Two Chinese strains isolated before the outbreaks, 606 and 1941, contain a portion (Region II, SSU05_0917~0933) of the 89K fragment (Figure 2 &3). A set of primers based on the sequence of Region II was designed (additional file 2), and the PCRs with primer pairs P2/P4, P3/P4, P5/P6, P7/P8, and P7/P9 all generated products of the expected sizes, indicating that the virulent strains 606 and 1941 contain Region II (Figure 4C). However, a ~1.5-kb amplicon was also obtained with primer pair P1/P17, suggesting that Region II in strains 606 and 1941 is found at different locations with respective to the 89K locus in the genome of 05ZYH33 (Figure 4C).

Virulent strain 8011 and avirulent strain T15, isolated in the Netherlands, appeared to have a similar polymorphism pattern in the 89K island. Both contain Region IV (SSU05_0936~0942 in strain 8011 and SSU05_0938~0942 in strain T15) and Region VI (SSU05_0961~0982) of the 89K element. This was evident from the NimbleGen CGS analysis (Figure 2 &3) and confirmed by further PCR analysis (Figure 4D &4E). PCR amplifications using primer pairs P10/P12, P11/P12, and P13/P14 for strain 8011, P10/P12, P11/P12, and P13/P14 for strain T15, and P15/P16 and P15/P17 for both strains yielded specific products of the expected sizes. The main difference between these two strains is the sequence that connects Region IV with VI. A ~1.5-kb fragment in strain 8011 (Figure 4D) and a 4.5-kb band in T15 (Figure 4E) was obtained by PCR using primers P13/P14, respectively. As in the case of strains 606 and 1941, Region IV and VI in strains 8011 and T15 also reside at different locations than the 89K island in 05ZYH33 because a 1.5-kb PCR product was detected by primer pair P1/P17 in both strains (Figure 4D &4E).

Avirulent strains 05HAS68 and ZF contain the first three genes (SSU05_0903~0905) of the 3'-end of the 89K PAI (Figure 2 &3). Further, strains 05HAS68, ZF, and 1941 appear to possess gene homologs to the Tn5252-like transposon region (Region VI, SSU05_0961~0982), though the sequences are less similar to that of the reference strain (Figure 3), which has been confirmed by shotgun sequencing of 05HAS68 (unpublished data). Together, our data suggest that the entire 89K island only exists in SS2 strains involved in the two outbreaks in China, and portions of the 89K element can be present in other SS2 strains. Additionally, the 89K sequence is highly polymorphic among different SS2 strains, and after thorough analysis, our results indicate that Region III (SSU05_0934~0935), Region V (SSU05_0943~0960), and the majority of Region I (SSU05_0906~0916) are exclusively present in STSS-causing SS2 strains.

### Deletions and significantly variant genes

In addition to the variations of the 89K PAI, the entire SS2 genome exhibits considerable diversity among different strains (Figure 2). Compared to the reference 05ZYH33 genome, the Chinese STSS-causing strains contain no obvious deletions or significant gene variances, which is consistent with their epidemiology and clinical presentation. For two strains (606 and 1941) isolated in China, but before the two STSS outbreaks, and another two strains (8011 and S10) isolated in the Netherlands, there are no detectable deletions or significantly variant genes, except for the 89K polymorphisms described above. However, four major deletions were identified in the other virulent and avirulent strains (Figure 2). As shown in Table 2, the virulent strain SS2-N lacks all four regions of differences (RDs), while strains 607 and S735 lack RD1, RD3, and RD4. RD4 is also absent in virulent strain 1940. Avirulent strain T15 is devoid of RD1 and RD3, and strains 05HAS68 and ZF lack RD3 and RD4. The four deletions were all characterized as having direct repeats and deviating GC content at their terminal sequences. Furthermore, RD3 and RD4 contain mobile genetic elements, such as recombinase and integrase, suggesting that they were acquired horizontally by strains with these regions present in their genome. Nevertheless, none of these regions carries known or putative virulence factors.

**Table 2:** Polymorphisms in the regions of differences (RDs) in the tested SS2 strains

RD	Genome position		Direct Repeat	GC content*	Absence in strains	Gene	Annotation
						
	Start	End					
RD1	93902	99882	99 bp	34.64%	607	SSU05_0100	unknown protein
					S735	SSU05_0101	unknown protein
					SS2-N	SSU05_0102	hypothetical protein
					T15	SSU05_0103	hypothetical protein
						SSU05_0104	unknown protein
						SSU05_0105	hypothetical protein
						SSU05_0106	hypothetical protein
						SSU05_0107	hypothetical protein

RD2	478619	481517	7 bp	42.64%	SS2-N	SSU05_0490	conserved hypothetical protein
						SSU05_0491	hydrolase (MutT family)
						SSU05_0492	ATP-binding subunit

RD3	834923	846186	15 bp	30.71%	05HAS68	SSU05_0862	site-specific recombinase
					ZF	SSU05_0863	hypothetical protein
					607	SSU05_0864	replication initiator protein
					S735	SSU05_0865	hypothetical protein
					SS2-N	SSU05_0866	hypothetical protein
					T15	SSU05_0867	hypothetical protein
						SSU05_0868	hypothetical protein
						SSU05_0869	hypothetical protein
						SSU05_0870	hypothetical protein
						SSU05_0871	hypothetical protein
						SSU05_0872	hypothetical cytosolic protein

RD4	1452704	1463847	19 bp	36.95%	05HAS68	SSU05_1513	site-specific recombinase
					1940	SSU05_1514	virulence-associated protein E
					ZF	SSU05_1515	hypothetical protein
					607	SSU05_1516	hypothetical protein
					SS2-N	SSU05_1517	hypothetical protein
					S735	SSU05_1518	hypothetical protein
						SSU05_1519	unknown protein
						SSU05_1520	unknown phage protein
						SSU05_1521	hypothetical protein
						SSU05_1522	hypothetical protein
						SSU05_1523	hypothetical protein
						SSU05_1524	hypothetical protein
						SSU05_1525	hypothetical protein
						SSU05_1526	transcriptional regulator
						SSU05_1527	transcriptional regulator
						SSU05_1528	Gp21 protein
						SSU05_1529	integrase
						SSU05_1530	integrase

* The average GC content of the 05ZYH33 chromosome is 41.1%.

Compared to the virulent strains, the three avirulent strains 05HAS68, ZF, and T15 contain additional deletions. A total of 194 genes are absent or display significant sequence variation compared to the reference strain (Figure 2). Among these 194 genes, 80 encode hypothetical proteins, and the remaining 114 encode proteins that are mainly involved in ABC-type transporters, phosphotransferase systems, transcription regulators, carbohydrate metabolism, co-factor biosynthesis, restriction-modification systems, and cellular surface structure. Strains 05HAS68 and ZF exhibit a similar deletion profile, except for three genes (SSU05_0216~0218) that are present only in ZF. Strains 05HAS68 and T15 exhibit 164 and 119 gene deletions or significantly variant genes, respectively, including 88 genes shared by all three avirulent strains (additional file 3). The virulence-related *epf *gene cluster [10,11], which encodes extracellular protein factor (EF; a putative virulence marker that widely exists in virulent strains) was absent in the avirulent strains [12]. Consistent with previous research, this gene cluster is present in all of the analyzed virulent strains. The orphan response regulator RevS proved to be important for SS2 pathogenesis was also absent in all three avirulent strains [13]. The roles of other genes specifically absent in these avirulent strains in virulence remain unknown. Some of them may be candidate virulence-related genes, and future research will focus on the possible role of these genes in the molecular pathogenesis of SS2.

### Single nucleotide polymorphisms (SNPs) in SS2 strains

**Figure 5:**
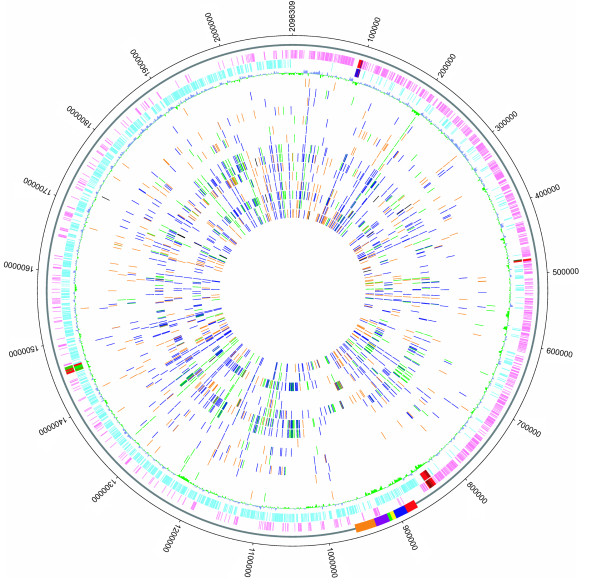
**Circular map of the identified SNPs relative to the 05ZYH33 chromosome**. The five outer circles are the same as Figure 2. Inner circles show all synonymous SNPs (green), non-synonymous SNPs (blue), premature stop codon SNPs (black), and intergenic SNPs (orange) identified in the 15 virulent strains tested: 98T003, Habb, JZD-1, ZYH214, ZYH214, ZYH354-1, JR, SS2-N, S735, 8011, S10, 606, 607, 1940, and 1941 from the sixth circle to the twentieth circle, respectively.

Aside from the detected deletions and significantly variant genes, 23 to 627 SNPs were identified in each of the virulent SS2 strains, but no SNPs were found in the avirulent strains compared to the 05ZYH33 reference strain (Figure 5). Roche NimbleGen tiling CGS arrays are sensitive to SNP detection only when the genomic divergence between the tested and the reference strains is < 0.5%. Thus, the lack of detected SNPs in the avirulent strains can be ascribed to the large genomic variations between these avirulent strains, which have been confirmed by shotgun-sequencing of strain 05HAS68 (unpublished data). On the whole, the identified SNPs in the STSS-causing virulent strains are significantly less abundant than those in the other virulent isolates. This result is consistent with the phylogenetic profiling described above, as well as the epidemiology and clinical presentation of these strains. Analysis of these SNPs is ongoing and is beginning to provide some explanation for the observed deletions and significantly variant genes.

## Discussion

In this study, Roche NimbleGen tiling arrays were used to detect the genomic diversity among epidemiologically distinct SS2 strains. One significant finding is the identification of specific genes that are only present in the STSS-causing strains involved in the two SS2 outbreaks in China. Previous work suggests that the 89K PAI is unique to SS2 strains involved in the STSS cases [6]. However, whole genome CGS analysis of 18 SS2 strains allowed us to carefully examine this island and identify specific regions that are present in the STSS-causing strains but not in any other SS2 strains.

We found that portions of the 89K fragment, particularly Regions II, IV, and VI, also exist in some other virulent and avirulent strains, albeit at different locations. Therefore, this finding cautions against the sole use of PCR amplification to evaluate the absence of the 89K PAI. Importantly, we found that Regions III, V, and part of I are exclusively present in the STSS-causing strains. Recently published work suggests that SS2 strain BM407, which was isolated from a human meningitis case in Vietnam, contains two regions with extended similarity to 89K but lacks most Region V genes [7]. These results indicate that genes within Region V likely contribute to the high invasiveness of the STSS-causing strains.

DNA sequencing analysis revealed that Region V encodes several ABC-type transporters (SSU05_0945~0949) and regulatory proteins, such as the SalK/R two-component system (SSU05_0943/0944), which is essential for full virulence of SS2 strains involved in the outbreaks in China (though its precise regulatory function remains unclear) [14]. One of the key questions arising from the two Chinese SS2 outbreaks is what bacterial factors contribute to the STSS that occurred in many patients. Phenotypic analysis ruled out the implication of super-antigens in the pathogenesis of STSS [15], and genome analysis has thus far failed to uncover any homologues of M protein, suggesting that alternative virulence mechanisms might account for the development of STSS. The ABC transport system (SSU05_0945~0949) encoded by Region V belongs to a large family of ABC transporters that export cell waste products or toxins, surface components, proteins, and other molecules involved in bacterial pathogenesis (e.g., hemolysin, heme-binding proteins, proteases, and peptide antibiotics) [16]. Therefore, participation of this ABC transport system in the pathogenesis of STSS cannot be excluded and requires further investigation.

Comparative analysis of the 18 SS2 strains also identified a number of genes, including several well-characterized virulence genes, that are absent in the three avirulent isolates, which may account for the loss of virulence and provide new insight into the virulence mechanisms of SS2. Among these absent gene products, PlcR is a major virulence regulator that was first described in the Gram-positive *Bacillus *group (including *B. cereus, B. thuringiensis*, and *B. anthracis*) [17], which activates the expression of toxins, hemolysins, phospholipases, proteases, bacteriocins, transporters, cell wall biogenesis, two-component sensors, and chemotaxis [18]. Until now, there were no reports about the role of PlcR in the *Streptococcus *genus. However, homologues of PlcR are ubiquitous in *S. pneumoniae, S. thermophilus*, and *S. equi*, indicating that this regulator may be functional in these organisms and a candidate virulence regulator in SS2.

In addition, lipase is important for bacteria to grow in carbohydrate-restricted conditions, where lipids are the sole carbon source, and help bacteria to stick to each other or to specific host tissues [19]. It has been well documented that lipase plays a key role in the colonization, dissemination, and adhesion of *Staphylococcus aureus *[20], *Staphylococcus epidermidis *[21], and *Pseudomonas aeruginosa *[22]. Based on these observations, it was reasonable to assume that lipase (SSU05_0325) is also related to the virulence of SS2 strains.

Sortases are responsible for the cleavage of many surface proteins characterized by a C-terminal LPXTG motif and the linkage of the processed proteins to the peptidoglycan layer of the cell wall of Gram-positive bacteria. In the *S. suis *05ZYH33 genome, sortase genes are distributed into four clusters: *srtA, srtBCD, srtE*, and *srtF*, respectively [23]. SrtA is involved in the localization of muramidase-released protein (MRP) and surface antigen one (Sao) [23,24]. Disruption of *srtA *impairs the colonizing potential in specific organs and attenuates the full virulence of *S. suis *[23,25]. Very recently, the *srtF *cluster was proven to be essential for pilus biosynthesis in SS2 [26]. The *srtBCD *cluster (SSU05_2096~2103) encodes nine proteins, including three sortases, three pilus subunits, and three ancillary proteins [23]. The *srtBCD *homologues in *S. pneumoniae *mediate the assembly and surface topology of adhesive pili, and deletion of the *srtBCD *cluster completely prevents pneumococcal pilus biogenesis [27]. Pili have recently been recognized in Gram-positive pathogens as important virulence factors involved in adhesion and invasion [28,29], and they increase pathogenicity in animal models [30,31]. Furthermore, genetic evidence also demonstrates that the pili of Gram-positive bacteria are antigenic and protective of immunized animals [28,29,32]. Given these facts, the absence of *srtBCD *cluster from the avirulent strains tested in our study strongly indicated that the pilus is important for SS2 pathogenesis and could be exploited as an immune target for SS2 infections.

For survival, bacteria have developed a variety of highly sophisticated and sensitive signal transduction pathways with which they adapt their gene expression patterns to meet the challenges of their ever-changing surroundings [33]. These mechanisms enable bacterial cells to communicate with their hosts, external environment, and between one another, allowing them to establish specific responses or develop specialized structures (e.g., biofilms) to ensure their survival, colonization, and dissemination. In this study, six putative transcriptional regulators and a response regulator (RevS; SSU05_2090) in signal transduction pathways were absent from the avirulent strains. RevS is an orphan response regulator and plays a role in the pathogenesis of SS2 infections [13]. The CtsR (SSU05_1976) regulator negatively regulates the expression of class III heat shock genes, which are required for stress survival, including growth at high temperature [34]. Although the other transcriptional regulators remain to be studied, it is reasonable to hypothesize that they may also be implicated in the pathogenesis of SS2.

Intriguingly, nearly all of the genes (81 of 88) missing in the three avirulent strains are also absent in SS2 strain 89/1591, based on the draft genome sequence of 89/1591 (additional file 3). Strain 89/1591 was isolated from a diseased pig and shown to be virulent in an experimental infection model [35,36]. It represents a typical North American virulent strain that lacks three virulence protein markers (MRP, EF, and suilysin). Here, we found that 89/1591 also lacks the candidate virulence genes that are described above, which are typically found in the European and Chinese virulent strains. Why is strain 89/1591 still virulent despite the lack of the virulence genes described above? This dilemma may be explained by the divergent evolution of 89/1591. The genome of 89/1591 is significantly different from the sequenced genomes of Chinese strains 05ZYH33 and 98HAH12, as well as European strain P1/7, containing deletions and insertions of hundreds of genes (data not shown). Thus, it appears that strain 89/1591 has evolved differently and acquired distinct sets of genes that contribute to its virulence, which accounts for its distinct clinical presentation and epidemiology compared to the European and Asian strains.

Collectively, our analysis indicates that the SS2 genome is highly polymorphic, which may reflect the organism's host specificity and clinical manifestation and provides information on the evolution of this important pathogen. A recent comparative genomic analysis indicates that gene gain is the major evolutionary force of streptococcal species, among which *S. suis *is the greatest lineage [37]. It is apparent that during the course of evolution, SS2 strains are under the highest level of positive selection pressure and have acquired foreign genes, mainly through the mechanism of HGT (e.g., conjugative transposons), to better adapt to host environments. The emergence of a new, highly virulent SS2 strain capable of causing STSS during the two outbreaks in China is the latest example of such adaptive evolution. Moreover, massive genomic rearrangement is also attributable to the evolutionary driving force of *S. suis *strains, as evidenced by the shotgun sequencing of the avirulent strain 05HAS68, which shows that large genomic rearrangements occurred between avirulent and virulent SS2 strains (unpublished data).

**Figure 6:**
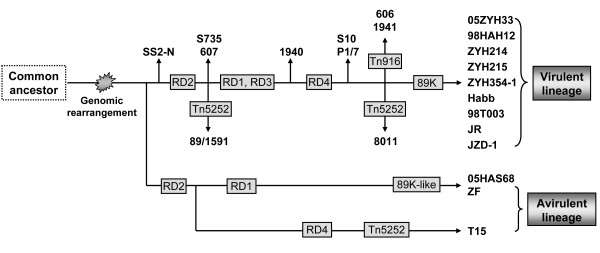
**Predicted evolutionary scenario of SS2 based on current knowledge of the genome data**. Polymorphisms in genomic regions of differences (RDs and 89 K) are indicated by gray boxes.

Based on the results of the current study, we propose a model of the microevolution of SS2 strains (Figure 6), which provides a framework for future investigations. Although the model remains to be confirmed, it is reasonable to assume that the common ancestor of SS2 underwent significant genomic rearrangements to form avirulent and virulent populations for the colonization and exploitation of novel niches. Thereafter, the avirulent and virulent lineages acquired divergent genes to improve their abilities to survive and evolve independently into the currently prevailing strains.

## Conclusions

In this work, significant genomic polymorphisms in SS2 populations were uncovered by the Roche NimbleGen CGS technique followed by PCR analysis, despite the fact that this DNA microarray-based method can only detect known gene sequences. The virulent strains isolated in China during the two STSS outbreaks are highly homologous, and the other virulent and avirulent strains are significantly polymorphic, which is consistent with the epidemiology and clinical presentation of these strains. The 89K fragment, a candidate PAI in the Chinese STSS-causing isolates, was found to be partially included in the other virulent and avirulent strains. In total, only 15 genes in the 89K PAI are unique to the STSS-causing strains. The ABC-type export systems encoded by these genes may represent an alternative virulence mechanism for the catastrophic features of toxic shock. In addition, a few presumptive virulence genes present in the virulent but not avirulent lineage were characterized. The presence of PlcR, lipase, sortases, the pilus proteins, the RevS response regulator, the CtsR regulator, and other transcriptional regulators may be important in the pathogenicity of SS2.

Our data also indicate that SS2 strains have highly divergent genomes. During evolution, SS2 strains have acquired many genes of foreign origin, mainly via mobile genetic elements, to better adapt to host environments. Moreover, massive genomic rearrangement appears to be another evolutional force in SS2 strains. Based on the current study, we propose a model of the microevolution of SS2 strains, which provides a framework for future investigations. This whole-genome comparative analysis has provided new insight into the pathogenicity and evolution of SS2 and may contribute to the development of potential therapies and novel preventive measures to control SS2 infections.

## Methods

### Bacterial strains, DNA extraction, and labeling

The *S. suis *strains used in this study are listed in Table 1. The virulent strains were isolated from diseased patients or swine, and the avirulent strains were isolated from healthy swine and were identified as avirulent in a pig model [4,12,14]. Cells were cultured in Todd-Hewitt broth (THB) (Difco Laboratories, Detroit, MI) medium. Genomic DNA was extracted using a QIAGEN Genomic-tip 100/G kit and labeled via random priming reactions. DNA (~1 μg) was mixed with 1 OD of 5'-fluorescent dye-labeled random nonamer (Cy3 for the SS2 strains analyzed and Cy5 for the 05ZYH33 reference strain) (TriLink Biotechnologies) in 62.5 mM Tris-HCl, 6.25 mM MgCl_2_, and 0.0875% β-mercaptoethanol, denatured at 98°C for 5 min, chilled on ice, and incubated with 100 units Klenow fragment (NEB) and dNTP mix (6 mM each in TE) for 2 h at 37°C. Reactions were terminated with 0.5 M EDTA (pH 8.0), precipitated with isopropanol, and resuspended in water. A 50-fold amplification was typically achieved.

### Mutation mapping microarray design

Mutation mapping microarrays were designed with NimbleGen algorithms that select a 29-mer oligonucleotide every seven bases on each strand of the reference genome sequence (GenBank Accession NC_009442). All probes were synthesized in parallel on a two-array set using a Digital Light Processor ™ (Texas Instruments, Plano Texas) and photoprotected phosphoramidite chemistry (i.e., maskless array synthesis) (NimbleGen Systems, Madison WI) in a random probe layout [38,39].

### Microarray hybridization

Labeled genomic DNA was hybridized to arrays in NimbleGen hybridization buffer for 16 h at 42°C using a MAUI hybridization system (BioMicro Systems, Inc. Salt Lake City, Utah). Labeled genomic DNA (~5 μg) from the 05ZYH33 reference strain and from each tested strain were co-hybridized to each array. Arrays were washed with NimbleGen wash buffer, spun dry in a high-speed microarray centrifuge (TeleChem International, Inc., Sunnyvale, CA), and stored until they were scanned.

### Analysis of mapping array data and design and hybridization of re-sequencing microarrays

Microarrays were scanned at 5-μm resolution using a Genepix^® ^4000B scanner (Axon Instruments, Union City CA), and pixel intensities were extracted using NimbleScan™ v2.4 software (NimbleGen). Probes that spanned potential mutations were identified by NimbleGen software. Probe sequences corresponding to all possible candidate mutation sites were selected for re-sequencing. The strategy used to automatically generate the sequencing arrays was similar to that described previously [9]. Briefly, eight probes were designed per base position analyzed, i.e., four per genome strand. These probes contain all possible alleles at a centrally located position. The length, melting temperature, and mismatch position of each probe were optimized. When target DNA is hybridized to these arrays, the perfectly matched probe will hybridize more strongly than the three corresponding mismatch probes for each strand. This differential signal intensity between the perfect match probe and mismatch probes allows the base to be determined precisely. These re-sequencing arrays were synthesized, hybridized with labeled genomic DNA from each tested SS2 strain, and scanned as above. Sequence base assignments were made using a machine-learning algorithm [40]. Putative mutation-containing DNA segments were PCR amplified and verified by capillary sequencing. The microarray data was deposited in the National Center for Biotechnology Information Gene Expression Omnibus Database (GEO; http://www.ncbi.nlm.nih.gov/geo/), with the accession number of GSE17868.

### Phylogenetic profile analysis

According to NimbleGen algorithms, each probe was assigned a binary profile (1 or 0) based on the probe signal intensity ratio (reference/test) to evaluate its presence/absence across different strains. The gene profiles were then clustered, and the strains were grouped based on the similarity of gene profiles. A maximum parsimony tree for SS2 strains was constructed using PHYLIP version 3.68 http://evolution.genetics.washington.edu/phylip.html based on the results of a bootstrapping test of strain phylogeny. The phylogenetic tree was visualized using TREEVIEW.

## Authors' contributions

BZ, JT, and GFG designed the study. ZW, ML, CW, JL, NL, RZ, and HL performed the experiments. ZW, ML, YJ, RY, and CL analyzed the data. BZ oversaw the experiments and wrote the manuscript. All authors read and approved the final manuscript.

## Supplementary Material

Additional file 1 **Gene content and annotation of the 89 K PAI**. Click here for file

Additional file 2 **Primers used for PCR confirmation of the 89 K polymorphisms in SS2 strains**. Click here for file

Additional file 3 **Deletions or significantly variant genes common to all three avirulent strains (05HAS68, ZF and T15) and their presence in strain 89/1591**. Click here for file

## References

[B1] Staats JJ Feder I Okwumabua O Chengappa MM *Streptococcus suis*: past and present Vet Res Commun 1997 21 6 381 407 10.1023/A:1005870317757 9266659

[B2] Feng Y Zhang H Ma Y Gao GF Uncovering newly emerging variants of *Streptococcus suis*, an important zoonotic agent Trends Microbiol 2010 18 3 124 131 10.1016/j.tim.2009.12.003 20071175

[B3] Ma Y Feng Y Liu D Gao GF Avian influenza virus, *Streptococcus suis *serotype 2, severe acute respiratory syndrome-coronavirus and beyond: molecular epidemiology, ecology and the situation in China Philos Trans R Soc Lond B Biol Sci 2009 364 1530 2725 2737 10.1098/rstb.2009.0093 19687041 PMC2865088

[B4] Tang J Wang C Feng Y Yang W Song H Chen Z Yu H Pan X Zhou X Wang H Streptococcal toxic shock syndrome caused by *Streptococcus suis *serotype 2 PLoS Med 2006 3 e151 10.1371/journal.pmed.0030151 16584289 PMC1434494

[B5] Li M Shen X Yan J Han H Zheng B Liu D Cheng H Zhao Y Rao X Wang C GI-type T4SS-mediated horizontal transfer of the 89 K pathogenicity island in epidemic *Streptococcus suis *serotype 2 Mol Microbiol 2011 79 6 1670 1683 10.1111/j.1365-2958.2011.07553.x 21244532 PMC3132442

[B6] Chen C Tang J Dong W Wang C Feng Y Wang J Zheng F Pan X Liu D Li M A Glimpse of Streptococcal Toxic Shock Syndrome from Comparative Genomics of *S. suis *2 Chinese Isolates PLoS ONE 2007 2 e315 10.1371/journal.pone.0000315 17375201 PMC1820848

[B7] Holden MT Hauser H Sanders M Ngo TH Cherevach I Cronin A Goodhead I Mungall K Quail MA Price C Rapid evolution of virulence and drug resistance in the emerging zoonotic pathogen *Streptococcus suis* PLoS One 2009 4 7 e6072 10.1371/journal.pone.0006072 19603075 PMC2705793

[B8] Leung AS Tran V Wu Z Yu X Alexander DC Gao GF Zhu B Liu J Novel genome polymorphisms in BCG vaccine strains and impact on efficacy BMC Genomics 2008 9 413 10.1186/1471-2164-9-413 18793412 PMC2553098

[B9] Wong CW Albert TJ Vega VB Norton JE Cutler DJ Richmond TA Stanton LW Liu ET Miller LD Tracking the evolution of the SARS coronavirus using high-throughput, high-density resequencing arrays Genome Res 2004 14 3 398 405 10.1101/gr.2141004 14993206 PMC353227

[B10] Berthelot-Herault F Morvan H Keribin AM Gottschalk M Kobisch M Production of muraminidase-released protein (MRP), extracellular factor (EF) and suilysin by field isolates of *Streptococcus suis *capsular types 2, 1/2, 9, 7 and 3 isolated from swine in France Vet Res 2000 31 5 473 479 10.1051/vetres:2000133 11050742

[B11] Smith HE Reek FH Vecht U Gielkens AL Smits MA Repeats in an extracellular protein of weakly pathogenic strains of *Streptococcus suis *type 2 are absent in pathogenic strains Infect Immun 1993 61 8 3318 3326 8335363 10.1128/iai.61.8.3318-3326.1993 PMC281006

[B12] Vecht U Wisselink HJ Jellema ML Smith HE Identification of two proteins associated with virulence of *Streptococcus suis *type 2 Infect Immun 1991 59 9 3156 3162 1879937 10.1128/iai.59.9.3156-3162.1991 PMC258147

[B13] de Greeff A Buys H van Alphen L Smith HE Response regulator important in pathogenesis of *Streptococcus suis *serotype 2 Microb Pathog 2002 33 4 185 192 12385746 10.1016/s0882-4010(02)90526-7

[B14] Li M Wang C Feng Y Pan X Cheng G Wang J Ge J Zheng F Cao M Dong Y SalK/SalR, a two-component signal transduction system, is essential for full virulence of highly invasive *Streptococcus suis *serotype 2 PLoS One 2008 3 5 e2080 10.1371/journal.pone.0002080 18461172 PMC2358977

[B15] Sriskandan S Slater JD Invasive disease and toxic shock due to zoonotic *Streptococcus suis*: an emerging infection in the East? PLoS Med 2006 3 5 e187 10.1371/journal.pmed.0030187 16594733 PMC1434506

[B16] Davidson AL Dassa E Orelle C Chen J Structure, function, and evolution of bacterial ATP-binding cassette systems Microbiol Mol Biol Rev 2008 72 2 317 364 10.1128/MMBR.00031-07 18535149 PMC2415747

[B17] Declerck N Bouillaut L Chaix D Rugani N Slamti L Hoh F Lereclus D Arold ST Structure of PlcR: Insights into virulence regulation and evolution of quorum sensing in Gram-positive bacteria Proc Natl Acad Sci USA 2007 104 47 18490 18495 10.1073/pnas.0704501104 17998541 PMC2141804

[B18] Gohar M Faegri K Perchat S Ravnum S Okstad OA Gominet M Kolsto AB Lereclus D The PlcR virulence regulon of *Bacillus cereus* PLoS One 2008 3 7 e2793 10.1371/journal.pone.0002793 18665214 PMC2464732

[B19] Hasan F Shah AA Hameed A Methods for detection and characterization of lipases: A comprehensive review Biotechnol Adv 2009 27 6 782 798 10.1016/j.biotechadv.2009.06.001 19539743

[B20] Rollof J Hedstrom SA Nilsson-Ehle P Lipolytic activity of *Staphylococcus aureus *strains from disseminated and localized infections Acta Pathol Microbiol Immunol Scand B 1987 95 2 109 113 3591310 10.1111/j.1699-0463.1987.tb03096.x

[B21] Longshaw CM Farrell AM Wright JD Holland KT Identification of a second lipase gene, *gehD*, in *Staphylococcus epidermidis*: comparison of sequence with those of other staphylococcal lipases Microbiology 2000 146 1419 1427 10846220 10.1099/00221287-146-6-1419

[B22] Wilhelm S Tommassen J Jaeger KE A novel lipolytic enzyme located in the outer membrane of *Pseudomonas aeruginosa* J Bacteriol 1999 181 22 6977 6986 10559163 10.1128/jb.181.22.6977-6986.1999 PMC94172

[B23] Wang C Li M Feng Y Zheng F Dong Y Pan X Cheng G Dong R Hu D Feng X The involvement of sortase A in high virulence of STSS-causing *Streptococcus suis *serotype 2 Arch Microbiol 2009 191 1 23 33 10.1007/s00203-008-0425-z 18716756

[B24] Osaki M Takamatsu D Shimoji Y Sekizaki T Characterization of *Streptococcus suis *genes encoding proteins homologous to sortase of gram-positive bacteria J Bacteriol 2002 184 4 971 982 10.1128/jb.184.4.971-982.2002 11807057 PMC134807

[B25] Vanier G Sekizaki T Dominguez-Punaro MC Esgleas M Osaki M Takamatsu D Segura M Gottschalk M Disruption of *srtA *gene in *Streptococcus suis *results in decreased interactions with endothelial cells and extracellular matrix proteins Vet Microbiol 2008 127 3-4 417 424 10.1016/j.vetmic.2007.08.032 17954016

[B26] Fittipaldi N Takamatsu D de la Cruz Dominguez-Punaro M Lecours MP Montpetit D Osaki M Sekizaki T Gottschalk M Mutations in the gene encoding the ancillary pilin subunit of the *Streptococcus suis srtF *cluster result in pili formed by the major subunit only PLoS One 5 1 e8426 10.1371/journal.pone.0008426 PMC2797073 20052283

[B27] Falker S Nelson AL Morfeldt E Jonas K Hultenby K Ries J Melefors O Normark S Henriques-Normark B Sortase-mediated assembly and surface topology of adhesive pneumococcal pili Mol Microbiol 2008 70 3 595 607 10.1111/j.1365-2958.2008.06396.x 18761697 PMC2680257

[B28] Mora M Bensi G Capo S Falugi F Zingaretti C Manetti AG Maggi T Taddei AR Grandi G Telford JL Group A Streptococcus produce pilus-like structures containing protective antigens and Lancefield T antigens Proc Natl Acad Sci USA 2005 102 43 15641 15646 10.1073/pnas.0507808102 16223875 PMC1253647

[B29] Rosini R Rinaudo CD Soriani M Lauer P Mora M Maione D Taddei A Santi I Ghezzo C Brettoni C Identification of novel genomic islands coding for antigenic pilus-like structures in *Streptococcus agalactiae* Mol Microbiol 2006 61 1 126 141 10.1111/j.1365-2958.2006.05225.x 16824100

[B30] Hava DL Camilli A Large-scale identification of serotype 4 *Streptococcus pneumoniae *virulence factors Mol Microbiol 2002 45 5 1389 1406 12207705 PMC2788772

[B31] Abbot EL Smith WD Siou GP Chiriboga C Smith RJ Wilson JA Hirst BH Kehoe MA Pili mediate specific adhesion of *Streptococcus pyogenes *to human tonsil and skin Cell Microbiol 2007 9 7 1822 1833 10.1111/j.1462-5822.2007.00918.x 17359232

[B32] Gianfaldoni C Censini S Hilleringmann M Moschioni M Facciotti C Pansegrau W Masignani V Covacci A Rappuoli R Barocchi MA *Streptococcus pneumoniae *pilus subunits protect mice against lethal challenge Infect Immun 2007 75 2 1059 1062 10.1128/IAI.01400-06 17145945 PMC1828493

[B33] Dubrac S Msadek T Tearing down the wall: peptidoglycan metabolism and the WalK/WalR (YycG/YycF) essential two-component system Adv Exp Med Biol 2008 631 214 228 10.1007/978-0-387-78885-2_15 18792692

[B34] Derre I Rapoport G Msadek T The CtsR regulator of stress response is active as a dimer and specifically degraded in vivo at 37 degrees C Mol Microbiol 2000 38 2 335 347 10.1046/j.1365-2958.2000.02124.x 11069659

[B35] Quessy S Dubreuil JD Caya M Higgins R Discrimination of virulent and avirulent *Streptococcus suis *capsular type 2 isolates from different geographical origins Infect Immun 1995 63 5 1975 1979 7729910 10.1128/iai.63.5.1975-1979.1995 PMC173252

[B36] Berthelot-Herault F Gottschalk M Morvan H Kobisch M Dilemma of virulence of *Streptococcus suis*: Canadian isolate 89-1591 characterized as a virulent strain using a standardized experimental model in pigs Can J Vet Res 2005 69 3 236 240 16187555 PMC1176304

[B37] Lefebure T Stanhope MJ Evolution of the core and pan-genome of *Streptococcus*: positive selection, recombination, and genome composition Genome Biol 2007 8 5 R71 10.1186/gb-2007-8-5-r71 17475002 PMC1929146

[B38] Nuwaysir EF Huang W Albert TJ Singh J Nuwaysir K Pitas A Richmond T Gorski T Berg JP Ballin J Gene expression analysis using oligonucleotide arrays produced by maskless photolithography Genome Res 2002 12 11 1749 1755 10.1101/gr.362402 12421762 PMC187555

[B39] Albert TJ Norton J Ott M Richmond T Nuwaysir K Nuwaysir EF Stengele KP Green RD Light-directed 5'-- > 3' synthesis of complex oligonucleotide microarrays Nucleic Acids Res 2003 31 7 e35 10.1093/nar/gng035 12655023 PMC152820

[B40] Molla M Shavlik J Richmond T Smith S A self-tuning method for one-chip SNP identification Proc IEEE Comput Syst Bioinform Conf 2004 69 79 10.1109/csb.2004.1332419 16448001

