# Supplementary material for: Probing genomic diversity and evolution of Streptococcus suis serotype 2 by NimbleGen tiling arrays
Source: BMC Genomics. 2011 May 10;12:219. doi: 10.1186/1471-2164-12-219 (PMC3118785; doi:10.1186/1471-2164-12-219)
Supplement: Additional file 1 — Gene content and annotation of the 89 K PAI. [file 1471-2164-12-219-S1.DOC]

**Table S1.** **Gene content and annotation of the 89K PAI.**

| **Gene** | **Anotation** | **Region** |
| --- | --- | --- |
| SSU05_0903 | site-specific recombinase, phage integrase family | I |
| SSU05_0904 | hypothetical protein |  |
| SSU05_0905 | transcriptional regulator, Cro/CI family |  |
| SSU05_0906 | NisK |  |
| SSU05_0907 | NisR |  |
| SSU05_0908 | hypothetic ABC membrane transporter |  |
| SSU05_0909 | ABC transporter integral membrane subunit |  |
| SSU05_0910 | ABC multidrug transporter |  |
| SSU05_0911 | ABC transporter, NBP/MSD fusion protein |  |
| SSU05_0912 | putative asparagine synthase |  |
| SSU05_0913 | Tn5252, relaxase |  |
| SSU05_0914 | Tn5252, Orf 9 protein |  |
| SSU05_0915 | Tn5252, ORF 10 protein |  |
| SSU05_0916 | putative Abi-alpha protein |  |
| SSU05_0917 | Tn916, transposase | II |
| SSU05_0918 | hypothetical protein |  |
| SSU05_0919 | hypothetical protein |  |
| SSU05_0920 | Tn916, transcriptional regulator, putative |  |
| SSU05_0922 | Translation elongation factor (GTPases) |  |
| SSU05_0921 | hypothetical protein |  |
| SSU05_0923 | Tn916, hypothetical protein |  |
| SSU05_0924 | hypothetical protein |  |
| SSU05_0925 | hypothetical protein |  |
| SSU05_0926 | hypothetical protein |  |
| SSU05_0927 | hypothetical protein |  |
| SSU05_0928 | hypothetical protein |  |
| SSU05_0929 | hypothetical protein |  |
| SSU05_0930 | Tn916, transcriptional regulator, putative |  |
| SSU05_0931 | hypothetical protein |  |
| SSU05_0932 | hypothetical protein |  |
| SSU05_0933 | hypothetical protein |  |
| SSU05_0934 | DNA helicase | III |
| SSU05_0935 | hypothetical protein |  |
| SSU05_0936 | Signal recognition particle GTPase | IV |
| SSU05_0937 | Predicted transcriptional regulator |  |
| SSU05_0938 | ATPases with chaperone activity, ATP-binding subunit |  |
| SSU05_0939 | Methyl-accepting chemotaxis protein |  |
| SSU05_0940 | hypothetical protein |  |
| SSU05_0941 | DNA primase (type) |  |
| SSU05_0942 | hypothetical protein |  |
| SSU05_0943 | SalK | V |
| SSU05_0944 | SalR |  |
| SSU05_0945 | hypothetic protein, putative membrane protein |  |
| SSU05_0946 | ABC transporter ATP-binding protein |  |
| SSU05_0947 | ABC-type multidrug transport system |  |
| SSU05_0948 | ABC-type bacteriocin/lantibiotic exporters |  |
| SSU05_0949 | lantibiotic modifying enzyme |  |
| SSU05_0950 | hypothetic protein |  |
| SSU05_0951 | DNA recombinase |  |
| SSU05_0952 | DNA recombinase |  |
| SSU05_0953 | DNA recombinase |  |
| SSU05_0954 | hypothetic protein |  |
| SSU05_0955 | DNA polymerase, beta-like region |  |
| SSU05_0956 | methyltransferase |  |
| SSU05_0957 | putative aminoglycoside 6-adenylyltansferase |  |
| SSU05_0958 | putative adenine phosphoribosyltransferase |  |
| SSU05_0959 | transposase |  |
| SSU05_0960 | lantibiotic mersacidin modifying enzyme |  |
| SSU05_0961 | hypothetical protein | VI |
| SSU05_0962 | SNF2 family protein |  |
| SSU05_0963 | hypothetical protein |  |
| SSU05_0964 | hypothetical protein |  |
| SSU05_0965 | agglutinin receptor |  |
| SSU05_0966 | predicted transcriptional regulator |  |
| SSU05_0967 | hypothetical protein |  |
| SSU05_0968 | Tn5252, Orf28 |  |
| SSU05_0969 | type IV secretory pathway, VirB4 component |  |
| SSU05_0970 | hypothetical protein |  |
| SSU05_0971 | ABC-type cobalt transport system |  |
| SSU05_0972 | hypothetical protein |  |
| SSU05_0973 | type IV secretory pathway, VirD4 component |  |
| SSU05_0974 | hypothetical protein |  |
| SSU05_0975 | protease, putative |  |
| SSU05_0976 | hypothetical protein |  |
| SSU05_0977 | arsenate reductase and related proteins, glutaredoxin family |  |
| SSU05_0978 | hypothetical protein |  |
| SSU05_0979 | C-5 cytosine-specific DNA methylase |  |
| SSU05_0980 | hypothetical protein |  |
| SSU05_0981 | hypothetical protein |  |
| SSU05_0982 | hypothetical protein |  |
