# Supplementary material for: Probing genomic diversity and evolution of Streptococcus suis serotype 2 by NimbleGen tiling arrays
Source: BMC Genomics. 2011 May 10;12:219. doi: 10.1186/1471-2164-12-219 (PMC3118785; doi:10.1186/1471-2164-12-219)
Supplement: Additional file 2 — Primers used for PCR confirmation of the 89 K polymorphisms in SS2 strains. [file 1471-2164-12-219-S2.DOC]

**Table S2.** **Primers used for PCR confirmation of the 89K polymorphisms in SS2 strains.**

| **Primer** | **Primer sequence (5’-3’)** | **Position in 05ZYH33 genome** |
| --- | --- | --- |
| P1 | CACGCATCTCGTAGAGTTTGAC | 871777-871798 |
| P2 | CCGACTTAAATATCAAAGAGCAG | 887906-887928 |
| P3 | GCTGTCAGAAGTGGTAAATAAGTAGT | 888383-888408 |
| P4 | CACTTAGAGAGAAAATCGCAGAG | 889445-889467 |
| P5 | GCTTCGCAATGGTTTTTCG | 896855-896873 |
| P6 | GATTTTGGTTCTTGGGGTTTA | 898135-898155 |
| P7 | TGTCGTCAGCATGTAAAAGGTA | 905646-905667 |
| P8 | TCAAAAGGTCGTTCCCCACT | 906003-906022 |
| P9 | ATGGATGGAGGATGTCAGATATA | 908077-908099 |
| P10 | GGATTTCTTGGTTGATTTGGGTT | 909913-909935 |
| P11 | AATCAAGGTGCTGAGATAGGACA | 910900-910922 |
| P12 | GTTACAATGGCTATTTAGACGGT | 911412-911434 |
| P13 | TGTCGGAAACTTTGGAGTAGGA | 914157-914178 |
| P14 | CGGAGTAGTAATGCCATTGACC | 934537-934558 |
| P15 | TGCCTCTCCTCCTCGTCCT | 960920-960938 |
| P16 | TCTGCCCCTTTTTTTGCGT | 961633-961651 |
| P17 | GCGTAGCTGCTTAGTGCTACAA | 962218-962239 |
