# Supplementary material for: Probing genomic diversity and evolution of Streptococcus suis serotype 2 by NimbleGen tiling arrays
Source: BMC Genomics. 2011 May 10;12:219. doi: 10.1186/1471-2164-12-219 (PMC3118785; doi:10.1186/1471-2164-12-219)
Supplement: Additional file 3 — Deletions or significantly variant genes common to all three avirulent strains (05HAS68, ZF and T15) and their presence in strain 89/1591. [file 1471-2164-12-219-S3.DOC]

**Table S3. Deletions or significantly variant genes common to all three avirulent strains (05HAS68, ZF and T15) and their presence in strain 89/1591.**

| **Gene** | **89/1591** | **Annotation** |
| --- | --- | --- |
| SSU05_0036 | – | hypothetical protein |
| SSU05_0037 | – | hypothetical protein |
| SSU05_0038 | – | hypothetical protein |
| SSU05_0177 | – | extracellular protein (EF cluster, ORF1) |
| SSU05_0178 | – | Epf-like protein (EF cluster, ORF2) |
| SSU05_0179 | – | putative RTX family exoprotein A gene (EF cluster, ORF3) |
| SSU05_0241 | – | transcriptional regulator PlcR, putative |
| SSU05_0242 | – | transcriptional regulator PlcR, putative |
| SSU05_0323 | – | hypothetical protein |
| SSU05_0325 | – | lipase |
| SSU05_0357 | – | deoxyguanosine triphosphate triphosphohydrolase-related protein |
| SSU05_0358 | – | deoxyguanosine triphosphate triphosphohydrolase-related protein |
| SSU05_0376 | – | hypothetical protein |
| SSU05_0377 | – | hypothetical protein |
| SSU05_0380 | – | putative transcriptional regulator |
| SSU05_0381 | – | hypothetical protein |
| SSU05_0382 | – | hypothetical protein |
| SSU05_0383 | – | putative ATP-binding protein |
| SSU05_0384 | – | hypothetical protein |
| SSU05_0385 | – | hypothetical protein |
| SSU05_0386 | – | hypothetical protein |
| SSU05_0387 | – | hypothetical protein |
| SSU05_0460 | – | hypothetical protein |
| SSU05_0461 | – | hypothetical protein |
| SSU05_0463 | – | DNA or RNA helicase of superfamily II |
| SSU05_0464 | – | hypothetical protein |
| SSU05_0465 | – | transcriptional regulator |
| SSU05_0697 | – | hypothetical protein |
| SSU05_0698 | – | hypothetical protein |
| SSU05_0699 | – | hypothetical protein |
| SSU05_0732 | – | predicted phosphatase/phosphohexomutase |
| SSU05_0733 | – | phosphomethylpyrimidine kinase |
| SSU05_0734 | – | hydroxyethylthiazole kinase |
| SSU05_0735 | – | thiamine-phosphate pyrophosphorylase |
| SSU05_0736 | – | hypothetical protein |
| SSU05_0737 | – | probable uridine phosphorylase |
| SSU05_0738 | – | uridine phosphorylase |
| SSU05_0739 | – | hypothetical protein |
| SSU05_0740 | – | cobalt ABC transporter permease protein |
| SSU05_0741 | – | cobalt ABC transporter ATP-binding protein |
| SSU05_0742 | – | cobalt ABC transporter ATP-binding protein |
| SSU05_0753 | – | muramidase-released protein (MRP) |
| SSU05_0759 | – | ABC-type phosphate transport system, ATPase component |
| SSU05_0840 | – | hypothetical protein |
| SSU05_0899 | – | hypothetical protein |
| SSU05_1227* | – | predicted metal-dependent membrane protease |
| SSU05_1228 | – | hypothetical protein |
| SSU05_1280* | + | glycosyltransferases involved in cell wall biogenesis |
| SSU05_1355 | + | ATPase component of ABC transporters with duplicated ATPase domains |
| SSU05_1356 | – | ABC transporter, ATP-binding protein |
| SSU05_1450 | – | type I restriction enzyme EcoKI specificity protein (S protein) |
| SSU05_1451 | – | type I restriction-modification system, S subunit |
| SSU05_1452 | – | EcoE type I restriction modification enzyme M subunit |
| SSU05_1453 | – | EcoA type I restriction-modification enzyme R subunit |
| SSU05_1567* | + | predicted endonuclease containing a URI domain |
| SSU05_1568 | – | hypothetical protein |
| SSU05_1589 | – | hypothetical protein |
| SSU05_1590 | + | predicted transcriptional regulator |
| SSU05_1762 | – | hypothetical protein |
| SSU05_1786 | + | restriction endonuclease S subunit |
| SSU05_1817 | + | phosphotransferase system IIC component, glucose/maltose/N-acetylglucosamine-specific |
| SSU05_1971 | – | hypothetical protein |
| SSU05_1972 | – | Nicotinamide mononucleotide transporter |
| SSU05_1973 | – | hypothetical protein |
| SSU05_1974 | – | transcriptional regulator |
| SSU05_1975 | – | ATPases with chaperone activity, ATP-binding subunit |
| SSU05_1976 | – | transcriptional regulator CtsR |
| SSU05_1977 | – | putative bacterocin transport accessory protein, Bta |
| SSU05_1978 | – | hypothetical protein |
| SSU05_1996 | – | metallo cofactor biosynthesis protein |
| SSU05_1997 | + | predicted transcriptional regulator |
| SSU05_2024 | – | homocysteine methyltransferase |
| SSU05_2025 | – | amino acid permease |
| SSU05_2026 | – | amino acid permease |
| SSU05_2048 | – | hypothetical protein |
| SSU05_2049 | – | hypothetical protein |
| SSU05_2089 | – | hypothetical protein |
| SSU05_2090 | – | RevS |
| SSU05_2091 | – | hypothetical protein |
| SSU05_2096 | – | sortase-like protein (Srt D) |
| SSU05_2097 | – | sortase-like protein (Srt C) |
| SSU05_2098 | – | sortase-like protein (Srt B) |
| SSU05_2099 | – | backbone pilus subunit |
| SSU05_2100 | – | backbone pilus subunit |
| SSU05_2101 | – | backbone pilus subunit |
| SSU05_2102 | – | collagen adhesion protein |
| SSU05_2103 | – | collagen adhesion protein |
| SSU05_2104 | – | hypothetical protein |

* significantly variant genes
